# The effect of the 7DL-7Ae#1L·7Ae#1S translocation on the productivity and quality of spring bread wheat grain

**DOI:** 10.18699/VJGB-22-65

**Published:** 2022-10

**Authors:** S.N. Sibikeev, E.I. Gultyaeva, A.E. Druzhin, L.V. Andreeva

**Affiliations:** Federal Center of Agriculture Research of the South-East Region, Saratov, Russia; All-Russian Research Institute of Plant Protection, Pushkin, St. Petersburg, Russia; Federal Center of Agriculture Research of the South-East Region, Saratov, Russia; Federal Center of Agriculture Research of the South-East Region, Saratov, Russia

**Keywords:** bread wheat, translocation 7DL-7Ae#1L·7Ae#1S, analogue lines, eff iciency of the Lr29 gene, effect on grain productivity and bread-making quality, мягкая пшеница, транслокация 7DL-7Ae#1L·7Ae#1S, линии-аналоги, эффективность гена Lr29, влияние на продуктивность и качество зерна

## Abstract

The 7DL-7Ae#1L·7Ae#1S translocation with the Lr29 gene attracts the attention of bread wheat breeders by its effectiveness against Puccinia triticina. However, its impact on useful agronomic traits has been little studied. In this report, the prebreeding value of 7DL-7Ae#1L·7Ae#1S was studied in analogue lines (ALs) of spring bread wheat cultivars Saratovskaya 68 and Saratovskaya 70 during 2019–2021. The presence of the Lr29 gene was conf irmed by using molecular marker Lr29F24. The ALs with the Lr29 gene were highly resistant to P. triticina against a natural epiphytotics background and in laboratory conditions. 7DL-7Ae#1L·7Ae#1S in Saratovskaya 68 ALs reduced grain productivity in all years of research. On average, the decrease was 35 and 42 %, or in absolute f igures 1163 and 1039 against 1802 kg/ha in the cultivar-recipient. In Saratovskaya 70 ALs, there was a decrease in grain yield in 2019 and 2020, and there were no differences in 2021. On average, the decrease was 18 and 32 %, or in absolute f igures 1101 and 912 against 1342 kg/ha in the cultivar-recipient. The analogues of both cultivars showed a signif icant decrease in the weight of 1000 grains, which ranged from 14 to 20 % for Saratovskaya 68 and 17–18 % for Saratovskaya 70. An increase in the period of germination-earing was noted only in Saratovskaya 68 lines, which averaged 1.3 days. ALs of Saratovskaya 70 had no differences in this trait. 7DL-7Ae#1L·7Ae#1S did not affect plant height and lodging resistance in all ALs. Studies of the bread-making quality in lines with 7DL-7Ae#1L·7Ae#1S revealed a signif icant increase in grain protein and gluten content. As for the effect on the alveograph indicators, there were differences between ALs of both cultivars. While Saratovskaya 68 ALs had a decrease in elasticity and in the ratio of dough tenacity to the extensibility, Saratovskaya 70 lines had an increase in these indicators. All lines increased the f lour strength and the loaves volume, but while Saratovskaya 68 ALs had an increased porosity rating, Saratovskaya 70 ALs had the same rating as the recipient.

## Abstract (ru)

Транслокация 7DL-7Ae#1L·7Ae#1S с геном Lr29 от хромосомы 7Ae#1 пырея удлиненного (2n = 70) привлекает внимание селекционеров мягкой пшеницы эффективностью против возбудителя листовой ржавчины. Однако ее влияние на хозяйственно полезные показатели изучено недостаточно. В представленной статье исследована агрономическая ценность транслокации 7DL-7Ae#1L·7Ae#1S у аналогов яровой мягкой пшеницы сортов Саратовская 68 и Саратовская 70 в течение 2019–2021 гг. Наличие гена Lr29 у исследуемого материала было подтверждено с помощью ПЦР-анализа с маркером Lr29F24. Линии с геном Lr29 характеризовались высокой устойчивостью к Puccinia triticina как на фоне естественной эпифитотии, так и в лабораторных условиях. Транслокация 7DL-7Ae#1L·7Ae#1S у аналогов сорта Саратовская 68 снижала продуктивность зерна во все годы исследований. В среднем понижение составило 35 и 42 %, или в абсолютных цифрах 1163 и 1039 против 1802 кг/ га у сорта-реципиента. В то же время у линий-аналогов сорта Саратовская 70 отмечено понижение урожайности зерна в 2019 и 2020 гг. и не было отличий в 2021 г. В среднем понижение урожайности было 18 и 32 %, или в абсолютных цифрах 1101 и 912 против 1342 кг/ га у сорта-реципиента. У аналогов обоих сортов выявлено значимое понижение массы 1000 зерен, которое колебалось от 14 до 20 % на сорте Саратовская 68 и 17–18 % – на сорте Саратовская 70. Увеличение периода «всходы–колошение» обнаружено только у линий-аналогов сорта Саратовская 68, которое составило в среднем 1.3 суток. У аналога сорта Саратовская 70 различий по этому показателю не было. Транслокация 7DL-7Ae#1L·7Ae#1S не влияла на вы- соту растений и устойчивость к полеганию у всего набора линий. Исследования качества муки и хлеба у линий с транслокацией 7DL-7Ae#1L·7Ae#1S выявили значимое увеличение содержания зернового белка и клейковины. Од- нако наблюдались различия между линиями сортов Саратовская 68 и Саратовская 70 по влиянию на показатели альвеографа. Если у линий сорта Саратовская 68 отмечалось понижение упругости, отношения упругости теста к растяжимости, то у линий сорта Саратовская 70 эти показатели повышались. Все линии увеличивали силу муки и объем хлебцев, но если у линий сорта Саратовская 68 была повышенная хлебопекарная оценка, то линии сорта Саратовская 70 не отличались от реципиента.

## Introduction

The Lower Volga region of the Russian Federation is one of the main regions growing bread wheat. The main crops are in the Saratov and Volgograd regions. In 2020, in the Saratov region, the total area under bread wheat (winter and spring) amounted to 1,380,524 ha (http://srtv.gks.ru/storage/media bank/f2raAGzs). In the Volgograd region, the total area under bread wheat (winter and spring) amounted to 1,528,000 ha (https://volgastat.gks.ru/storage/mediabank/posev_21.pdf). According to “The State Register of Selection Achievements Authorized for Use for Production Purposes” in 2021, 90 cultivars of winter bread wheat and 27 cultivars of spring bread wheat have been registered in the Lower Volga region (gossort rf.ru/wp-content/uploads/2021/04/Final-register-2021.pdf).

In this region, one of the main fungal diseases of wheat is leaf rust (pathogen Puccinia triticina f. sp. tritici Erikss.). Despite the fact that some Russian grain-growing regions of the last decade are characterized by a decrease in the importance of this disease, the losses from it are quite large (Gultyaeva et al., 2021). In the Lower Volga region, the disease occurs annually, and strong epiphytoties are observed every three to four years (Gultyaeva et al., 2020). The last strong epiphytoty was in the growing season of 2017 (Sibikeev et al., 2020). Despite the above-mentioned large number of winter and spring bread wheat cultivars registered in this region, a significant part of them are susceptible to P. triticina (Gultyaeva et al., 2021). Thus, in the Left Bank zone of the Saratov region, the prevailing cultivars of spring bread wheat are Saratovskaya 42, Saratovskaya 55 and Albidum 32, which are not protected by any resistance genes or they have ineffective Lr10 (Gultyaeva et al., 2020, 2021).

In general, each region of Russia has its own set of common wheat cultivars with leaf rust resistance genes (Lr-genes). However, in general, it is not large and is limited to genes Lr1, Lr3, Lr9, Lr10, Lr19, Lr20, Lr24, Lr26, Lr34, Lr37 and Lr6Agi1, Lr6Agi2, LrSp. These genes are used in practical breeding in various combinations, but in general, only LrSp, Lr6Agi1, and Lr6Agi2 genes have not been overcome (Gultyaeva et al., 2021). Moreover, there is reason to believe that the last two genes are allelic (Sibikeev et al., 2017). In this regard, most breeding centers in Russia are searching for and transferring new unidentified Lr-genes from wild relatives into promising material (Davoyan et al., 2017, 2019, 2021; Gultyaeva et al., 2020) or attracting effective previously unused Lr-genes (Sibikeev et al., 2019). The latter include the Lr29 gene, which is highly effective both in Russia (Gultyaeva et al., 2021) and abroad (Labuschagne et al., 2002; Li et al., 2018; Atia et al., 2021).

As is known, the Lr29 gene is introgressed into the bread wheat cultivar Chinese Spring from the short arm 7Ae#1 of the chromosome Agropyrum elongatum (Host) Beauvois =Thinopyrum ponticum (Podp.) Backworth and Dewey by homeologous recombination (Sears, 1973). E.R. Sears (1973) identified a 7D/Ag#11 transfer that differed from others in its resistant response to the leaf rust pathogen. Unlike other leaf rust resistance genes (Lr24, Lr19) introduced from Ag. elongatum, the Lr29 gene is not linked to stem rust resistance genes and yellow flour (McIntosh et al., 1995). The catalog of wheat gene symbols does not list any commercial cultivars with this gene (McIntosh et al., 2013). However, there is information on the presence of Lr29 in Egyptian varieties (Atia et al., 2021). Based on the research of E.I. Gultyaeva, it is absent in Russian cultivars of winter and spring bread wheat (Gultyaeva et al., 2021). The reason for the limited use of the Lr29 gene in practical breeding, more precisely the 7DL-7Ae#1L·7Ae#1S translocation, is not known.

The question of whether this is due to the fact that it does not compensate for the absence of wheat chromatin, or contains undesirable linkages, is open, since there is little information on the effect of this translocation on economically useful traits. There are only two studies of the 7DL-7Ae#1L·7Ae#1S translocation available to us: they were conducted in Canada and South Africa, focused mainly on the study of flour and bread quality and were carried out in small plot crops for one or two growing seasons (Dyck, Lukow, 1988; Labuschagne et al., 2002). In Russia, such studies have not been conducted. To identify the effect of the 7DL-7Ae#1L·7Ae#1S translocation with the Lr29 gene on grain productivity and the quality of bread flour in the laboratory of genetics and cytology of the Federal Center of Agriculture Research of the South-East Region, analogue lines of spring bread wheat were bred using Saratovskaya 68 and Saratovskaya 70 cultivars

The purpose of our research was to reveal the prospects of the 7DL-7Ae#1L·7Ae#1S translocation with the Lr29 gene for practical breeding both in terms of effectiveness against P. triticina and in terms of its effect on grain productivity and flour and bread quality.

## Materials and methods

The material used included the following genotypes: 1) cultivar- recipient of spring bread wheat Saratovskaya 68 (С68) and Saratovskaya 70 (С70); 2) analogue lines of spring bread wheat Saratovskaya 68*4//TcLr29; 3) analogue lines of spring bread wheat Saratovskaya 70*4//TcLr29. Analogue lines were obtained by crossing the C68 and C70 cultivars with a near isogenic line of the Thatcher cultivar (TcLr29, RL-6080) containing the 7DL-7Ae#1L·7Ae#1S translocation with the Lr29 gene, followed by fourfold backcrossing with cultivarrecipients. In total, 20 analogue lines were obtained from the C68 cultivar and 11 lines were obtained from the C70 cultivar. For further studies, two lines of analogues for each cultivar were taken. Since both recipient cultivars are susceptible to the leaf rust pathogen, the main criterion for backcross selection was resistance to P. triticina.

Two different recipient cultivars were taken into the study to identify the possible influence of the recipient genotype on the studied traits. C68 and C70 cultivars differ from each other. The first cultivar is awned, red-grained, white-ears, tallgrowing, mid-ripening, susceptible to the leaf rust pathogen, contains the ineffective Lr10 gene (Gultyaeva et al., 2020), belongs to the category of valuable wheat in terms of flour and bread quality. The second cultivar is awnless, white-grained, white-ears, tall-growing, early maturing, susceptible to the leaf rust pathogen, does not contain any Lr-genes (Gultyaeva et al., 2020); belongs to the category of valuable wheat in terms of flour and bread quality.

The studies included three stages: the first stage was to confirm the presence of alien material in the studied analogue lines С68*4//TcLr29 (С68Lr29) and С70*4//TcLr29 (С70Lr29), Lr-genes were identified using the molecular marker Lr29 (Lr29F24) (Procunier et al., 1995). DNA was isolated from the leaves of 5-day-old seedlings by the micro method according to the method of D.V. Dorokhov and E. Clocke (Dorokhov, Clocke, 1997). Three plants were taken from each line. The DNA concentration in the standard solution was 50–100 ng/ μl. The polymerase chain reaction was carried out in a MyCycler Thermal Cycler (Bio-Rad, USA) under the following conditions: 94 °С – 3 min, 35 cycles (94 °С – 30 s; 60 °С – 30 s; 72 °С – 1 min). The amplified fragments were separated by electrophoresis in 1.5 % agarose gel in 1×TBE buffer; the gels were stained with ethidium bromide and photographed under ultraviolet light. The TcLr29 line was used as a positive control.

The second stage was an evaluation of the lines susceptibility to the pathogen of leaf rust at the juvenile stage and the stage of adult plants. The susceptibility of plant material at the stage of adult plants (milky-wax ripeness phase) was evaluated in the field conditions of the Federal Center of Agriculture Research of the South-East Region during a strong epiphytoty of the pathogen in 2017 (Sibikeev et al., 2020).

In the field, the resistance degree was determined using the A.P. Roelfs et al. (1992) scale, where R is resistant, MR is moderately resistant, MS is moderately susceptible, and S is susceptible, respectively. The percentage degree of rust damage was assessed according to the scale of R.F. Peterson et al. (1948). Lines at the juvenile stage were evaluated in laboratory conditions in the first leaf phase at the All-Russian Institute of Plant Protection in 2018. P. triticina clones marked with virulence for genes Lr9 (K9), Lr19 (K19), Lr26 (K26) and the combined Saratov population of the pathogen were used. Test clone K9 was isolated from the Ural population, test clone K19 – from Tambov, K26 – from Krasnodar population, respectively. The Saratov population was collected at the Lysogorsk phytonursery of the Saratov region in 2018. The test clones and population used were avirulent to Thatcher (TcLr) lines with genes Lr24, Lr23, Lr28, Lr29, Lr39(= 41), Lr45, Lr47, Lr51, Lr53 and virulent to those with Lr1, Lr2a, Lr2b, Lr2c, Lr3a, Lr3bg, Lr3ka, Lr10, Lr14a, Lr15, Lr16, Lr17, Lr18, Lr20, Lr30.

Clone K9 was virulent to TcLr9 and avirulent to TcLr19, and TcLr26; clone K19 was virulent to TcLr19 and avirulent to TcLr9, TcLr26; clone K26 was virulent to TcLr26, avirulent to TcLr9, and TcLr19. These P. triticina test clones were chosen for analysis, since virulence to Lr9 is common in the Ural region, to Lr19 – in the Middle and Lower Volga regions, and to Lr26 – in all regions of the Russian Federation where bread wheat is grown.

The Saratov population of the pathogen was represented by a mixture of two races: virulent to the TcLr19 line, avirulent to TcLr9, TcLr26 and virulent to the TcLr26 line, avirulent to TcLr9, TcLr19. For infection, 10–12 day old seedlings (the first leaf phase) of the studied lines of analogues and recipient cultivars grown in pots with soil were used. They were sprayed with an aqueous suspension of spores of each test clone and a population with the addition of Tween 80 detergent. After infection, the plants were placed in a light installation under controlled conditions (temperature 20 °C, photoperiod 16 h day/8 h night). The type of wheat reaction was determined according to the scale of E.B. Mains, H.S. Jackson (1926), where 0 is the absence of symptoms; 0; – necrosis without pustules; 1 – very small pustules surrounded by necrosis; 2 – pustules of medium size, surrounded by necrosis or chlorosis; 3 – pustules of medium size without necrosis, 4 – large pustules without necrosis, X – pustules on the same leaf of different types, chlorosis and necrosis are present. Plants with 0–2 point damage were classified as resistant (R), and 3, 4 and X (S) were classified as susceptible (Mains, Jackson, 1926).

The third stage is the evaluation of grain productivity traits, physical and baking properties of dough and bread in the С68Lr29 and С70Lr29 analogue lines in comparison with the recipient cultivars С68 and С70. The studies were carried out in 2019–2021. The hydrothermal coefficient for the growing season of bread wheat in 2019 was 0.6 (very dry conditions), in 2020 – 0.8 (dry conditions) and 0.9 (dry conditions) in 2021. The main differences between the weather conditions in 2019 and 2021 there were high temperatures during the flowering period (above the long-term average by 4.2 and 8.0 °C, respectively) with a reduced amount of precipitation (below the long-term average by 13 mm), which sharply reduced grain productivity. At the same time, in 2020, during the flowering period, a lower temperature was observed (below the long-term average by 1 °C) with an increased amount of precipitation (above the long-term average by 48 mm), which increased the grain yield.

The experimental material was randomly sown in 7 m2 plots in three replications. The seeding rate was 400 grains per 1 m2. The bread-making quality was evaluated by the content of crude gluten, gluten strength and the indicators of the IDG-3 device (deformation index of gluten) and the Chopin alveograph with the baking of experimental bread samples. The protein content of grain harvested in 2020 and 2021 was determined on the Infratec™ 1241 Grain Analyzer. The data obtained for each set of lines of analogues and the corresponding recipient cultivars were subjected to one-way ANOVA with multiple comparisons according to Duncan using the Agros-2.10 breeding and genetic software package.

## Results

Identification of resistance genes

To confirm the presence of the 7DL-7Ae#1L·7Ae#1S translocation, and, accordingly, the Lr29 gene, PCR analysis with the Lr29F24 marker was performed in the C68Lr29 and C70Lr29 analogue lines (Procunier et al., 1995).

Amplification fragments, 900 bp in size, were detected in the entire set of C68Lr29 and C70Lr29 analogue lines, as well as in the positive control (TcLr29 line). 31 samples were analyzed; a 900 bp size amplification product was determined in lines No. 6, 8, 11, 14, 19, 20 – C68Lr29, No. 21, 31 – C70Lr29 (see the Figure). Based on the molecular analysis performed using a marker developed to detect the 7DL-7Ae#1L·7Ae#1S translocation with the Lr29 gene, it was suggested that the C68Lr29 and C70Lr29 analogue lines carry this translocation, and hence the Lr29 gene. To reveal the effect of the 7DL-7Ae#1L·7Ae#1S translocation on economically valuable traits, lines No. 6 and 8 C68Lr29 and lines No. 21, 31 C70Lr29 were chosen.

**Fig. 1.**
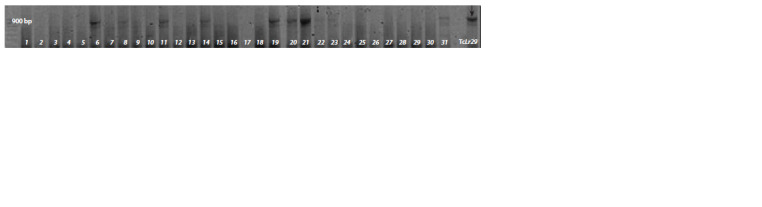
Electrophoregram of the fragments amplification in the presence of the Lr29F24/Lr29R24 marker. M – length marker of 1000/100–500 (Diam). The arrow indicates a 900 bp diagnostic fragment. Tracks 1–20 – analogue lines C68Lr29; 21–31 – analogue lines С70Lr29.

Phytopathological analysis of resistance to the leaf rust causative agent

Under the leaf rust epiphytotiсs condition of 2017, all lines with the Lr29 gene had a resistant reaction type (R) (infestation 0 %, reaction type – IT = 11+), while the recipient cultivars C68 and C70 had susceptibility to the pathogen (S) (infestation 40 and 60 %, reaction type IT = 3). Similar results were obtained during lines inoculation in the seedling phase in laboratory conditions (Table 1).

**Table 1.**
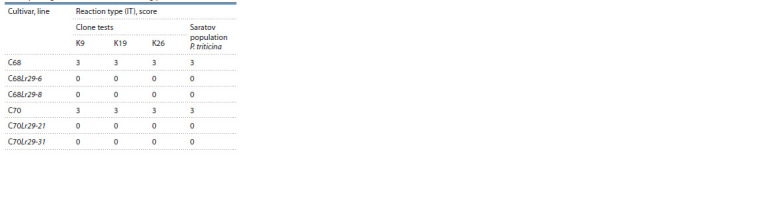
Characteristics of lines susceptibility with translocation 7DL-7Ae#1L·7Ae#1S and parental cultivars to the pathogen P. triticina in the seedling phase

Thus, phytopathological analysis of resistance to the leaf rust pathogen in the C68Lr29 and C70Lr29 analogue lines under field and laboratory conditions evidenced a high level of their resistance and the effectiveness of the Lr29 gene, compared with the original recipient cultivars.

Effect of the 7DL-7Ae#1L·7Ae#1S translocation on grain productivity and flour and bread quality

The results of studying grain productivity in lines with the 7DL-7Ae#1L·7Ae#1S translocation (Lr29 gene) showed that, on average, for the period from 2019 to 2021, there are no significant differences in yield in the lines compared to the recipient cultivars C68 and C70 (Table 2). This is expected, since the grain productivity traits in 2020 are three to five times higher than the grain yield in 2019 and 2021. Similar results were obtained when identifying the effect of Sr22+Sr25 and Sr22+Sr35 gene combinations on lines of spring bread wheat compared to the L503 and Favorit cultivars. The grain yield of these cultivars and lines was 2.3–2.7 times higher in 2020 compared to 2019 (Sibikeev et al., 2021). Nevertheless, the analysis of grain productivity separately by years revealed that, in the C68Lr29 analogue lines, for all three years of study, the grain yield was significantly lower than that of the recipient cultivar C68. Similar conclusions were reached when comparing the grain productivity of the C70Lr29 lines for two years of study (2019 and 2020), and only in the growing season of 2021 the grain productivity of the lines was at the level of the recipient cultivar C70.

**Table 2.**
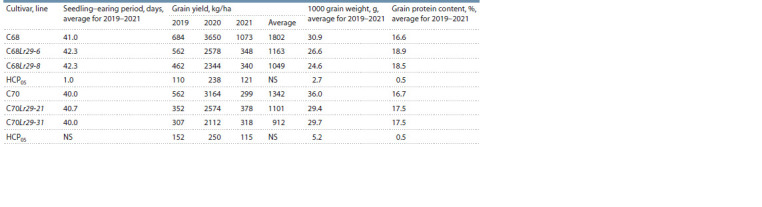
Grain productivity traits in spring bread wheat lines with the translocation 7DL-7Ae#1L·7Ae#1S (Lr29 gene) in 2019–2021

The 2019–2021 seasons were characterized by drought, but the 2020 season was distinguished by precipitation distribution during the growing season. This year, there was moisture excess from germination to flowering, and then there was a drought with high temperatures until full maturity. The main positive moment of the growing season in 2020 was the increased precipitation amount in the third decade of June (the flowering phase of spring bread wheat). At the same time, the excess of long-term indicators was 80 % at low air temperatures, which further contributed to a higher grain yield.

On average for 2019–2021, the analysis of the 1000 grain weight, as one of the important elements of grain productivity, showed a significant decrease in the С68Lr29 lines – 26.6 and 24.6 g compared to the recipient cultivar – 30.9 g. Similar results were obtained for the C70Lr29 analogue lines – 29.4 and 29.7 g versus 36.0 g for C70 (see Table 2).

On average for 2019–2021, the effect of the 7DL-7Ae#1L· 7Ae#1S (Lr29 gene) translocation on the duration of the germination-earing period was ambiguous. If significant differences were observed between the C68Lr29 lines (42.3 days) and the recipient cultivar C68 (41.0 days), then there were no differences between the C70Lr29 lines (40.7 and 40.0 days) and the cultivar C70 (40.0 days). Thus, the effect of the 7DL-7Ae#1L·7Ae#1S translocation was not the same in different genotypes of the recipient cultivars: in lines based on the mid-season cultivar C68, the germination-earing period lengthened, and on the early-ripening cultivar C70, it remained at the recipient level. There were no differences in plant height and lodging resistance between the studied lines and the recipient cultivars.

Unfortunately, the involvement of alien genetic variability in the bread wheat gene pool worsens some traits of flour and bread quality. Therefore, when studying the effect of chromosome introgression or translocations from related species into bread wheat, an important step is to determine the quality of the final product – flour and bread. On average for 2020–2021, studies revealed that lines with the 7DL-7Ae#1L·7Ae#1S translocation (Lr29 gene) significantly exceeded the recipient cultivars in grain protein content (see Table 2). Moreover, the C68Lr29 analogue lines exceeded the recipient cultivars by 2 %, and C70Lr29 – by 0.8 %.

According to the indicators of gluten, the following results were obtained: the C68Lr29 lines significantly exceeded the recipient cultivar C68 in gluten content, namely 41.7 and 41.4 versus 31.4 % in the recipient, LSD05 = 2.5. There were no significant differences in gluten strength between the C68Lr29 lines and the recipient, but it should be noted that, according to IDK-3, the C68Lr29 lines have weaker gluten – 76 and 80 units, against 72 units in C68. The C70Lr29 lines showed a significant excess in gluten content of the recipient cultivar C70, namely 37.0 and 38.5 versus 35.0 % in the recipient, LSD05 = 1.5. There were no significant differences in gluten strength between the C70Lr29 lines and the recipient cultivar. In addition, according to the indicators of IDK-3, in the C70Lr29 lines, the gluten of the first group is 71 and 75 units, in C70 – 69 units, respectively.

When studying the alveograph indicators, it was found that the C68Lr29 lines differed not only from the recipient cultivar, but also from each other. According to the dough elasticity and the ratio of the dough tenacity to extensibility (P/L), there was a decrease, but in one of the C68Lr29 lines, the decrease in elasticity (P) was insignificant. The C68Lr29 lines showed an ambiguous effect of the 7DL-7Ae#1L·7Ae#1S translocation on the flour strength: one line slightly decreased, and the second one significantly increased this indicator. Crumb porosity and bread volume in the C68Lr29 lines increased relative to the recipient cultivar C68, but in one of the lines the bread volume increase was insignificant. At the same time, in the C70Lr29 lines, the effect of the 7DL-7Ae#1L·7Ae#1S translocation on the alveograph parameters was unambiguous: an increase in dough elasticity, equal to the P/L ratio, an increase in flour strength, bread volume, and a high score for bread porosity at the level of the recipient cultivar C70 (Table 3).

**Table 3.**
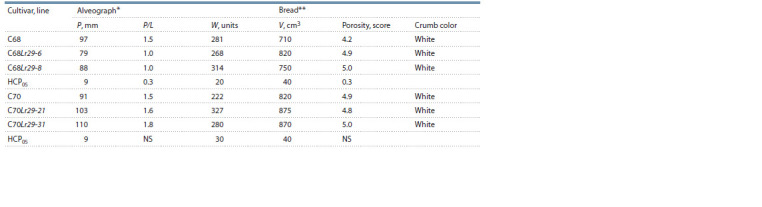
Bread-making quality traits in lines of spring bread wheat with the 7DL-7Ae#1L·7Ae#1S translocation (Lr29 gene) for 2020 on the average * Indicators of the alveograph: P – dough tenacity; P/L – tenacity to extensibility ratio; W – flour strength. ** Indicators of bread evaluation: V – bread volume; porosity.

## Discussion

As noted above, the Lr29 gene in the 7DL-7Ae#1L·7Ae#1S translocation is highly effective against leaf rust pathogen populations in many countries of the world. Only two P. triticina pathotypes from Turkey and one from Pakistan are known to be virulent to this gene (Huerta-Espino, 1992, from McIntosh et al., 1995). In our studies, the effectiveness of the Lr29 gene was confirmed during severe leaf rust epiphytosis in the Saratov region (R-type resistance and type of response to the pathogen IT = 1) and in laboratory studies. Lines with the Lr29 gene were resistant when inoculated with P. triticina isolates virulent to Lr9, Lr19, Lr26 (IT = 0;). Since, under field conditions, adult plants were evaluated in the phase of the beginning of grain filling, and in laboratory studies, seedlings were evaluated in the one leaf phase, it can be argued that the protective effect of Lr29 was expressed throughout the growing season.

Analyzing the effect of the 7DL-7Ae#1L·7Ae#1S translocation (gene Lr29), it is necessary to note the translocation size. As can be seen from its designation, it includes a part of the long arm and the entire short arm of the chromosome 7Ae#1 of Thinopyrum ponticum and a part of the long arm of the 7D chromosome of bread wheat. The break point is indicated at the distal part of 7DL-7Ae#1L of arms (Friebe et al., 1996). Thus, there is reason to expect a large impact on agronomic traits, primarily on grain productivity and the quality of flour and bread.

Unfortunately, there are few studies on the effect of the 7DL-7Ae#1L·7Ae#1S translocation (gene Lr29) on economically valuable traits (prebreeding study) (Dyck, Lukow, 1988; Labuschagne et al., 2002). These studies were carried out on near isogenic lines of the Thatcher cultivar (Dyck, Lukow, 1988) and Thatcher and Karee cultivars (Labuschagne et al., 2002). They mainly focused on determining the effect of the 7DL-7Ae#1L·7Ae#1S translocation (Lr29 gene) on breadmaking quality traits. Grain productivity has been studied during one year, and it showed a neutral effect (Dyck, Lukow, 1988).

In our studies, based on the results of three-year field trials under conditions of moisture deficiency (drought of varying degrees), a significant decrease in grain productivity was observed in the C68Lr29 lines for all three seasons. A similar effect was found in the C70Lr29 lines: a significant decrease in grain yield for two seasons out of three. P.L. Dyck, O.M. Lukow (1988) and M.T. Labuschagne et al. (2002) found a positive effect on grain protein content (Dyck, Lukow, 1988; Labuschagne et al., 2002). Our results are consistent with this conclusion. The increase in the grain protein content of the analogue lines compared to the recipient cultivars ranged from 0.8 to 2.0 %. The conclusions about a positive effect on the volume of experimental breads also coincided. According to the results of our studies, the excess was from 40 to 110 cm3. In the ratio of dough tenacity to extensibility (P/L), same as in the studies of M.T. Labuschagne et al. (2002), we determined the effect of the recipient variety. Thus, a decrease was noted on the С68Lr29 lines, and a neutral effect on the С70Lr29 lines. In studies by P.L. Dyck and O.M. Lukow (1988), a positive or neutral effect on the weight of 1000 grains was noted (Dyck, Lukow, 1988). According to our data, the presence of the 7DL-7Ae#1L·7Ae#1S translocation lowers this parameter, moreover, in two sets of analogue lines it decreases over three years of study. The decrease was from 4.3 to 6.6 g.

For the rest of the studied traits, our studies complement the results of P.L. Dyck, O.M. Lukow (1988) and M.T. Labuschagne et al. (2002). So, in the studies of P.L. Dyck, O.M. Lukow (1988) and M.T. Labuschagne et al. (2002), a positive or neutral effect on water absorption capacity and flour yield, and a negative effect on dough formation time were found. Our studies have established a positive effect on the gluten content and a slight decrease in its strength. In addition, the effect of the recipient cultivar on the dough elasticity was revealed, so in the С68Lr29 lines the 7DL-7Ae#1L·7Ae#1S translocation reduces this indicator, and in С70Lr29 it is significantly increased. In terms of the effect on the flour strength, the C70Lr29 lines showed a significant increase, while in the C68Lr29 lines, one line slightly decreased, and the second significantly increased this indicator. It is possible that, in addition to the effect of the 7DL-7Ae#1L·7Ae#1S translocation, the set of the C68Lr29 lines was also affected by selection during line generation. M.T. Labuschagne et al. (2002) also observed selection effects within a set of near isogenic lines of the Karee cultivar with the Lr29 gene, which had ambiguous flour quality indicators.

In our studies, all analogue lines either increased bread porosity indicators (C68Lr29 lines) or had high indicators at the level of the recipient cultivar (C70Lr29 lines). In addition, a different effect (depending on the recipient cultivar) on the duration of the seedling – earing period was revealed. Thus, significant differences were observed between the C68Lr29 lines (42.3 days) and the recipient cultivar C68 (41 days), while there were no differences between the C70Lr29 lines (40.7 and 40.0 days) and the variety C70 (40.0 days). No effect of the 7DL-7Ae#1L·7Ae#1S translocation on plant height and lodging resistance was found.

## Conclusion

The high efficiency of the Lr29 gene against the Saratov population of the leaf rust pathogen, as well as pathotypes virulent to Lr9, Lr19, Lr26, was confirmed. For the whole complex of economically valuable traits, analogue lines with the 7DL-7Ae#1L·7Ae#1S translocation (gene Lr29) were more promising than the recipient cultivars in terms of flour and bread quality, but were inferior to them in terms of grain productivity. The decrease in grain yield is apparently associated with a decrease in drought resistance compared to the recipient cultivars Saratovskaya 68 and Saratovskaya 70. For further use of the 7DL-7Ae#1L·7Ae#1S translocation (Lr29 gene) in breeding programs, additional studies are needed to reduce the negative impact on a number of agronomically important traits.

## Conflict of interest

The authors declare no conflict of interest.
